# Railway suicide in England and Wales 2000–2013: a time-trends analysis

**DOI:** 10.1186/s12889-016-2944-x

**Published:** 2016-03-15

**Authors:** Anna K. Taylor, Duleeka W. Knipe, Kyla H. Thomas

**Affiliations:** School of Social and Community Medicine, University of Bristol, Address-School of Social and Community Medicine, 39 Whatley Road, Bristol, BS8 2PS UK

**Keywords:** Suicide, Railways, England and Wales, Epidemiology, Train

## Abstract

**Background:**

In 2010, the “Tackling Suicide on the Railways” programme was launched as a joint initiative among Network Rail, the Samaritans and other key organisations such as the British Transport Police and train operators to achieve a 20 % reduction in railway suicides from 2010 to 2015 in Great Britain. We report the most recent age and sex specific trends in railway suicide in England and Wales from 2000 to 2013 and examine whether the initiative’s target reduction in railway suicides is likely to be achieved.

**Methods:**

Population data and suicide mortality data (all methods combined and railway) for England and Wales were obtained from the Office for National Statistics (ONS) and used to calculate age and gender specific rates for deaths registered from 2000 to 2013. Data on railway suicides were also obtained from the Rail Safety and Standards Board (RSSB) and compared with ONS data. We used joinpoint regression to identify changes in suicide trends across the study period.

**Results:**

The railway was used in 4.1 % of all suicides in England and Wales (RSSB data were similar to ONS data for most years). Suicides in all persons from all causes decreased from 2000 to 2007, with small increases from 2008 until 2013; this rise was entirely due to an increase in male suicides. Railway suicide rates increased over the entire study period; the proportion of railway suicides in all persons increased from 3.5 to 4.9 % during the study period. This trend was also mainly driven by increases in male suicides as female railway suicide rates remained steady over time. The highest age specific railway suicide rates were observed in middle aged men and women. Although there was no conclusive evidence of an increase in ONS railway suicides, RSSB data showed a statistically significant increase in railway suicides in males from 2009 onwards.

**Conclusion:**

The continued rise in male railway suicide in England and Wales is concerning, particularly due to the high economic costs and psychological trauma associated with these deaths. The initiative’s target of a 20 % reduction in railway suicide is unlikely to be achieved.

## Background

Railway suicide is a relatively uncommon method of suicide worldwide; between 1 and 10 % of all suicides involve the railway [1]. However, it is a highly lethal method of suicide, with a reported case fatality rate of 94 % [2]. Each railway suicide in the UK is estimated to cost approximately £61,000; the yearly financial cost to the rail industry was estimated to be £50 million in 2005 [3]. In addition to the high economic costs of train delays and other aspects of managing these fatalities, railway suicides may be associated with significant psychological effects on friends and relatives, train drivers, emergency personnel and onlookers [1, 4]. Therefore, the prevention of railway suicides is a priority for the rail industry.

In 2010, the “Tackling Suicide on the Railways” programme was launched as a joint initiative among Network Rail (the organisation responsible for rail infrastructure in Britain- www.networkrail.co.uk), the UK charity Samaritans (which provides emotional support to people who are emotionally distressed or experiencing suicidal thoughts - www.samaritans.org) and other organisations such as the British Transport Police and train operators [5]. The aim of the programme was to improve the railway industry’s knowledge of railway suicide and to reduce the number of railway suicides in Great Britain by 20 % from 2010 to 2015. A £5 million investment was made [6]. Several activities were implemented as part of the programme, including the use of posters to increase public awareness of the Samaritans, training for rail staff in how to manage suicidal contacts, trauma support training for staff affected by railway suicide and physical alterations such as mid platform fencing at railway stations [5].

There has been no recent analysis of time trends in railway suicide within the UK since Clarke [7] examined railway suicide in England and Wales from 1850 to 1949. The aim of this paper is to report the most recent age and sex-specific trends in railway suicide in England and Wales from 2000 to 2013 and to determine whether the “Tackling Suicide on the Railways” programme is likely to achieve its proposed target of a 20 % reduction in railway suicides.

## Methods

### Data sources

Data on confirmed suicides from all methods and the railway were obtained from the Office for National Statistics (ONS- http://www.ons.gov.uk) for deaths registered for England and Wales in the years 2000 to 2013. Confirmed suicides reported by the ONS were identified as such based on coroners’ verdicts (in the UK a coroner’s inquest is required for any death that is violent or unnatural, occurs under police custody or is sudden and of unknown cause) [8]. We included undetermined deaths (or open verdicts) in our suicide statistics as this is standard practice for the reporting of suicide statistics in England and Wales [9]. The International Classification of Diseases (ICD) codes were as follows: all suicides (ICD-9 E950-E959, E980-E989 excluding E988.8 and ICD-10 X60-X84, Y10-Y34 excluding Y33.9) and railway suicides (ICD-9 E958.0 and E988.0 and ICD-10 X81 and Y31). Mid-year population data for England and Wales were also obtained from the ONS from 2000 to 2013.

Data were also obtained from the Rail Safety and Standards Board (RSSB- www.rssb.co.uk) from 2000 to 2014 to estimate the numbers of railway suicides that occurred in a particular year in England and Wales [10]. The RSSB determined that a death was a railway suicide instead of an accidental fatality on the railway if the death was assessed as intentional based on the presence of one of the following criteria: (i) the presence of a suicide note, (ii) a clear statement of suicidal intent to an informant, (iii) behaviour which demonstrates suicidal intent, (iv) previous suicide attempts, (v) prolonged depression and (vi) the presence of emotional instability due to recent stress or evidence of failure to cope such as a breakdown [10].

### Statistical analysis

Stata version 13.0 (StataCorp, USA) and Excel 2013 (Microsoft, USA) were used for the statistical analyses. Yearly age standardised rates were calculated using the revised 2013 European Standard Population [11] for those aged 15 years and over by sex. Yearly age and sex specific rates were calculated for three age groups (<35 years, 35–64 years and ≥65 years). All suicide rates in the figures were expressed as numbers of deaths per million persons, men or women.

We used joinpoint regression to identify changes in the trends of yearly age standardised suicide rates for those aged 15 years and over across the study period (2000–2013 for ONS data, 2000–2014 for RSSB data). This method uses contiguous linear segments and join points (i.e. points at which trends change) to describe trends in mortality rates. We specified a maximum of two join points and fitted log-linear regression models to detect the number and location of join points and the annual percent change (APC) in rates between join points. The probability of type 1 error (i.e. concluding that one or more join points were present when no join points exist) was set at 0.05. We reported the results of the best fit joinpoint regression models. If join points were detected in the trends for those aged 15 years and over, we performed further joinpoint analyses to identify changes in the age specific trends (<35, 35–64 and over 65 years). Analyses were performed using the SEER*Stat joinpoint trend analysis software, version 4.2.0.2).

### Ethics approval

Ethics approval was not required for this study.

## Results

From 2000 to 2013 inclusive, 61,290 deaths were identified by the ONS as suicides in England and Wales, 46,206 in males and 15,084 in females; of these 2,517 (4.1 %) were railway suicides (1,985 (4.3 %) in males and 532 (3.5 %) in females). The numbers of railway suicides using ONS data and RSSB data are shown in Table 1. Similar numbers of suicides were reported from both sources with some exceptions (years 2005, 2006, 2009 and 2012). Only ONS data are reported in the sections on overall and age-specific suicide trends.

**Table 1:** Numbers of railway suicides in England and Wales from 2000 to 2014 using data from the Office for National Statistics (ONS) and the Rail Safety and Standards Board (RSSB)

	2000	2001	2002	2003	2004	2005	2006	2007	2008	2009	2010	2011	2012	2013	2014
Male RSSB	118	118	134	131	137	155	165	154	153	151	163	163	183	198	206
Male ONS	129	117	139	120	127	132	152	135	163	134	146	155	141	195	NA
Female RSSB	39	37	34	29	41	28	57	33	39	47	46	34	46	30	44
Female ONS	37	34	35	31	43	30	36	45	36	43	61	34	34	33	NA
Persons RSSB	157	155	168	160	178	183	222	187	192	198	209	197	229	228	250
Persons ONS	166	151	174	151	170	162	188	180	199	177	207	189	175	228	NA
Difference between RSSB and ONS recorded suicides (persons)	−9	4	−6	9	8	21	34	7	−7	21	2	8	54	0	NA

*NA* note ONS suicide data were not yet available for 2014 at the time of the study

### Overall suicide trends

**Fig. 1:**
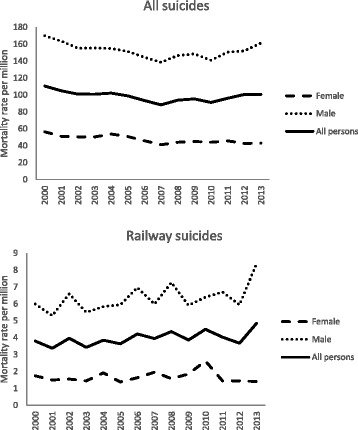
Age standardised suicide rates (all causes and railway) for ages ≥15 years in England and Wales, 2000–2013 (ONS data)

Figure 1 shows the age standardised suicide rates for males, females and all persons from all causes and the railway for those aged 15 years and over from 2000 to 2013. Suicide trends in all persons were mostly driven by the male suicide trends. In males, there was a general downward trend in overall age standardised suicide rates (all methods combined) from 169.9 per million in 2000 to 138.5 per million in 2007. However, rates increased by 16.5 % from 138.5 per million in 2007 to 161.3 per million in 2013. From 2010 to 2013 (i.e. the time period during which the Tackling Railway Suicide Partnership was in place), male suicide rates from all causes increased by 14.4 % from 141 per million to 161.3 million. Similar to males, overall female suicide rates showed a decreasing trend from 56.5 per million in 2000 to 41.1 per million in 2007. However, since 2007, overall age standardised female suicide rates have remained relatively stable ranging from 43 to 44 per million.

The male railway suicide rate increased from 6.0 per million in 2000 to 8.4 per million in 2013. From 2010 to 2013 male railway suicide rates increased by 31.3 % from 6.4 per million to 8.4 per million. Female railway suicide rates remained relatively steady at about 2 per million from 2000 to 2013, with the exception of a small peak in 2010 when suicide rates were closer to 3 per million. RSSB data showed an upward trend in male rail suicides from 2009 onwards and female rail suicides from 2013 to 2014 (Table 1).

**Fig. 2:**
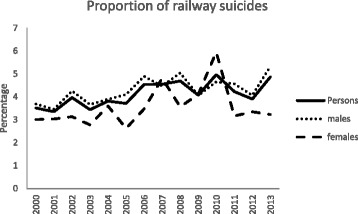
Railway suicides as a proportion of all suicides in England and Wales, 2000–2013 (ONS data)

Figure 2 shows the trends in railway suicide as a percentage of all suicides. In all persons, the proportion of railway suicides increased from 3.5 % in 2000 to 4.9 % in 2013. In men, the proportion of railway suicides increased from 3.7 to 5.3 % over the 14 year study period. In women, the proportion of railway suicides fluctuated over time; the highest proportion of railway suicides (6 %) was observed in 2010.

### Age specific suicide trends

**Fig. 3:**
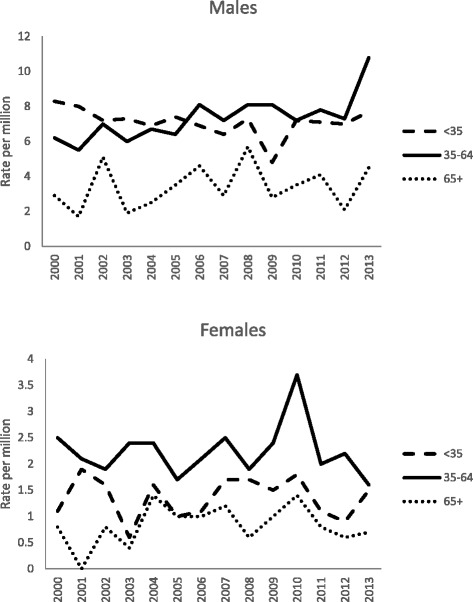
Age specific mortality rates for railway suicides in England and Wales, 2000–2013 (ONS data)

Figure 3 shows the age specific suicide rates for railway suicides from 2000 to 2013. Amongst males, the highest suicide rates were seen in those aged 35–64 years for most of the study period. The peak age specific suicide rate of 10.8 per million was observed in males aged 35–64 years in 2013. Males aged ≥65 years had the lowest rates of railway suicides across the entire time period. Women aged 35–64 years had the highest rates of railway suicides. Due to the small numbers involved there were wide fluctuations in the rates of railway suicides in the youngest and oldest women. However, similar to men, the lowest suicide rates were observed in the oldest women. Women in all age groups showed a peak in suicide rates in 2010; the highest age specific suicide rate of 3.7 per million was observed in women aged 35 to 64 years in 2010.

### Joinpoint regression analyses

Table 2 summarises the annual percent changes (APC) and join points (JPs) for suicide rates by gender for all causes (ONS data) and the railway (ONS and RSSB data). For suicides from all methods (using ONS data) the best fit model included one join point. There was evidence of a downward trend until 2007 in males aged 15 years and over (APC −2.5, 95 % CI −3.7 to −1.4) followed by an upward trend in suicide rates after 2007 (APC 2.3, 95 % CI 0.8 to 3.8). There was statistical evidence of a decreasing trend in suicides from all methods until 2006 in men aged <35 years (APC −6.2, 95 % CI −7.9 to −4.5) and an increasing trend in middle aged men (men aged 35–64 years) from 2007 onwards (APC 3.0, 95 % CI 1.7 to 4.4). Suicide rates from all causes remained stable in women.

**Table 2:** Summary of the annual percent changes (APCs) and join points (JPs) for trends in age standardised and age specific suicide rates by sex in England and Wales (2000–2013 ONS data and 2000–2014 RSSB data)

	Segment 1		Segment 2		Segment 3
	APC (95 % CI)	JP 1 (95 % CI)	APC (95 % CI)	JP 2 (95 % CI)	APC (95 % CI)
ONS Data					
*All suicides (1 join points for all persons and males; and no join point for females)* ^a^					
All persons	−2.6 (−3.6,-1.5)	2007 (2006,2010)	1.6 (0.2,3.0)		
Male	−2.5 (−3.7,-1.4)	2007 (2005,2011)	2.3 (0.8,3.8)		
Female	−3.0 (−4.6,-1.4)	2007 (2002,2011)	−0.8 (−3.0,1.4)		
*Rail suicides (No join points)*					
All persons	1.2 (−0.3,2.8)	2011 (2002,2011)	4.4 (−15.3,28.7)		
Male	1.0 (−1.0,3.0)	2011 (2002,2011)	9.8 (−15.8,43.2)		
Female	3.3 (0.4,6.2)	2010 (2008,2011)	−14,2 (−29.2,4.0)		
*Male All suicides by age group (1 join point for two youngest age groups and no join points for older men)* ^a^					
<35	−6.2 (−7.9,-4.5)	2006 (2005,2008)	−0.4 (−2.0,1.1)		
35-64	−0.8 (−1.9,0.3)	2007 (2005,2011)	3.0 (1.7,4.4)		
65 plus	−2.9 (−4.4,-1.4)	2009 (2002,2011)	1.6 (−3.5,7.0)		
RSSB Data					
*Rail suicides (No join point for all persons and females; and 2 join points for males)* ^a^					
All persons	4.8 (2.0,7.6)	2006 (2002,2009)	−2.4 (−16.0,13.3)	2009 (2005,2012)	4.4 (1.2,7.6)
Male	5.4 (3.3,7.5)	2006 (2004,2007)	−3.4 (−13.4,7.7)	2009 (2008,2012)	6.3 (3.9,8.7)
Female	−2.8 (−8.5,3.4)	2003 (2002,2009)	13.0 (−38.3,106.7)	2006 (2005,2012)	−2.8 (−8.5,3.4)
*Male Rail suicides by age group (No join points)* ^a^					
<35	1.7 (−8.9,13.6)	2005 (2002,2009)	−4.2 (−27.0,25.6)	2009 (2005,2012)	4.6 (−5.7,16.0)
35-64	16.0 (−22.1,72.7)	2002 (2002, 2009)	3.6 (0.6,6.7)	2012 (2005,2012)	−0.7 (−24.7,31.0)
65 plus	9.7 (−9.3,32.8)	2006 (2002,2009)	−7.0 (−47.2,63.8)	2010 (2005,2012)	12.7 (−12.3,44.8)

^a^ *The best fit join point model is described in italics*

There was no statistical evidence of changes in trends in railway suicide rates in males or females using ONS mortality data. However, when RSSB data were used, the best fit model included two join points and there was statistical evidence of an upward trend in railway suicide in men aged 15 years and over from 2000 to 2006 (APC 5.4, 95 % CI 3.3–7.5) and from 2009 onwards (APC 6.3, 95 % CI 3.9–8.7).

## Discussion

### Main findings

There are several key findings from this time trends analysis. First, over the 14 year time period (2000–2013), railway suicides accounted for only 4.1 % of all suicides (4.3 % in males and 3.5 % in females) using ONS data. Second, male suicide rates from all causes declined from 169.9 per million in 2000 to 138.5 per million in 2007, increasing to 161.3 per million in 2013; these findings were supported statistically by the join point analyses This contrasts with the consistent increase in railway suicide rates observed in males from 6.0 per million in 2000 to 9.9 per million in 2013; the proportion of railway suicides increased from 3.5 to 4.9 % over the duration of the study. However, there was no statistical evidence for this upward trend. In females, suicide rates (all causes and railway) remained steadier over time, although a peak in female railway suicide rates was observed in 2010. Third, the impact of the “Tackling Railway Suicides” programme on railway suicides is unclear. Male railway suicide rates increased by 31.3 % from 6.4 per million in 2010 to 8.4 per million in 2013. However female railway suicide rates almost halved from 2.6 per million in 2010 to 1.4 per million in 2011, with little change afterwards. RSSB data showed increases in the numbers of railway suicides in males from 2009 to 2014 and females from 2013 to 2014; the upward trend in male suicide rates using RSSB data was statistically significant. Last, the highest age specific railway suicide rates were observed in middle aged men and women.

### Strengths and limitations

This is the first study to examine 21st century railway suicide trends in England and Wales. Prior to this study, Clarke [7] examined earlier railway suicide trends in England and Wales from 1850 to 1949. We included undetermined deaths (or open verdicts) in our suicide statistics as this is standard practice for the reporting of suicide statistics in England and Wales [9]. However, there have been concerns about the reliability of national suicide statistics due to coroners’ increasing use of narrative verdicts [12, 13].

ONS data and RSSB data on railway suicides are not directly comparable. This is because ONS data are based on the year of registration of the suicide whereas RSSB data are based on the year of occurrence of the suicide. In addition, there can be long delays between the date of the suicide death and the date of registration of the suicide in the ONS. For example, the ONS reported that in 2013, the average registration delay for suicides was 168 days in England and 143 days in Wales [14]. A little over half of all suicides registered in England in 2013 had occurred in the previous years (38 % for Wales) [14]. However, it is reassuring that for the majority of the study period, the number of railway suicides obtained from ONS data was similar to the number reported by the RSSB data (Table 1). Last, we were unable to examine differences in suicide rates by region of death as these data were not available for this study.

### Comparison with other studies

#### Epidemiology of railway suicides

##### UK studies

The use of the railway is an uncommon method of suicide in the UK. In 2013, the most common method of suicide was hanging, strangulation and suffocation, which accounted for 56.1 % of male suicides and 40.2 % of female suicides [14]. In an earlier study by Clarke [7], the railway was used in approximately 5–6 % of male suicides and 3–4 % of female suicides in England and Wales. Similar percentages were observed in this current study for females (3.5 %), although the percentage for males was slightly lower (4.3 %). Another study which examined changes in the method of suicide from 1901 to 1907 to 2001 to 2007 found that the proportion of male railway suicide remained similar (4.9 % vs 4.7 %) [15]. However, the proportion of female railway suicide had increased from 2.2 to 4.4 % [15]. Like the Clarke [7] study, we found an excess of male railway suicides compared with female suicides. Additionally, in the earlier study, railway suicide was more common in the youngest age group (<35 years). This was not the case in the current study where the highest rates were seen in those aged 35–64 years.

##### European studies

The proportion of total suicides that occur on the railway varies considerably across Europe [16–18]. The proportion of railway suicide was higher in other Western European countries than Great Britain; it accounted for 5 % of all suicides in Belgium [16], 7 % of all suicides in Germany [18] and 11.5 % of all suicides in the Netherlands [17]. In addition, there were differences in the railway suicide trends. Whereas the incidence of railway suicide in all persons showed a more consistent increase over time in England and Wales, in Belgium, the incidence of railway suicide increased from 2000 to 2006, but declined afterwards from 2007 to 2009 [16]. In the Netherlands, the railway suicide rate increased from 11.4 per million in 2000 to 12.6 per million in 2001, declined to 10.5 per million in 2004 and increased afterwards to 11.8 per million in 2007 [17]. Post 2007 data were not available for the Netherlands [17]; the German study only reported data from 1991 to 2000 [18]. A more recent analysis of German railway suicides compared patterns of railway suicides in two observation periods (1995–1998 and 2005–2008), pre- and post-implementation of a German railway suicide prevention programme in 2002 [19]. Fewer suicides occurred in the latter observation period, however it was not possible to determine the impact of the intervention as age and sex-standardised rates were not reported [19].

#### Prevention of railway suicides

A recent systematic review examined the literature on the prevention of suicides and trespassing incidents on the railway [20]. A wide range of measures was reported including the use of fencing and other physical barriers (such as the use of platform screen doors), monitoring and detection systems (such as an automated security system which consisted of motion detectors, video cameras, infrared cameras and speakers), staff training to identify and approach people who may be at risk of suicide, use of lighting systems to influence behaviour (for example calming blue lights), enforcement (use of patrols in hotspots), availability of emergency information and outreach support at suicide hotspots and appropriate reporting or broadcasting of critical incidents [20]. However, the evidence for the effectiveness of these interventions was generally non-existent. Further research is needed to properly evaluate these measures before recommendations can be made.

## Conclusions

Railway suicides accounted for a relatively small proportion of all suicide deaths throughout the time period. There was conclusive evidence of an upward trend in male suicide rates for all suicide methods from 2007 using ONS data; however, there was no conclusive evidence of a rise in male railway suicides using ONS data. In contrast, we observed a statistically significant upward trend in male railway suicides from 2009 onwards using RSSB data. This discrepancy may be explained by the different methods used by the ONS and the RSSB to identify suicides. The rise in the use of railway suicide as a method of suicide in males is concerning, especially as these deaths are generally associated with significant economic costs and psychological trauma. There is a lack of evidence for a clear impact of the “Tackling Railway Suicide” programme on reducing railway suicide rates. It is unlikely that the original target of a 20 % reduction in railway suicide from 2010 to 2015 will be achieved.

## Availability of data and materials

Suicide data for England and Wales were available on request from the ONS and the RSSB. Population data were available from the ONS see https://www.ons.gov.uk/peoplepopulationandcommunity/populationandmigration/populationestimates.
